# Hearing and Listening Difficulties in High Schools and Universities: The Results of an Exploratory Survey of a Large Number of Students and Teachers in the Friuli-Venezia Giulia and Umbria Regions, Italy

**DOI:** 10.3390/audiolres15030066

**Published:** 2025-06-06

**Authors:** Valeria Gambacorta, Davide Stivalini, Niccolò Granieri, Raffaella Marchi, Alessia Fabbri, Pasquale Viola, Alessia Astorina, Ambra Fastelli, Giampietro Ricci, Eva Orzan

**Affiliations:** 1 Section of Otorhinolaryngology, Department of Medicine & Surgery, University of Perugia, 06126 Perugia, Italy; 2 Audiology and Otorhinolaryngology Unit, Institute for Maternal and Child Health IRCCS “Burlo Garofolo”, 34137 Trieste, Italy; 3 Unit of Audiology, Regional Centre for Cochlear Implants and ENT Diseases, Department of Experimental and Clinical Medicine, Magna Graecia University, 88100 Catanzaro, Italy

**Keywords:** acoustics, learning, hearing impairment, teachers, students, noise

## Abstract

Background/Objectives: with the aim of describing how students and their teachers perceive and define their hearing and auditory experience in the classroom, we present the results of a questionnaire that examined the listening challenges faced by students and teachers at the University of Perugia and in four secondary schools in Friuli-Venezia Giulia, Italy. Methods: A survey was developed as part of the A.Ba.Co. project (Overcoming Communication Barriers). Closed or open-ended questions were used to analyze the responses of students and teachers regarding diagnosed or only perceived hearing difficulties in daily life and the quality of listening in school classes. Results: Hearing difficulties, either clinically diagnosed or only perceived, were reported by 8–9% of students. Between teachers, the reported hearing difficulties were 27.1% in high school and 12% at university (*p* < 0.001). The most frequent reason for less-than-optimal ease of listening in class differed between the two educational levels; 45.8% of high school students blamed it on the noise in the room compared to 18.2% of university students (*p* < 0.001). Inversely, 40.9% of university students connected listening difficulty with their place in class compared to 9.5% (101/1065) of high school students (*p* < 0.001). Conclusions: Although the minimum acoustic requirements for educational facilities have been established by the UNI 11532-2 standard, it is speculated that the majority of high school and university classrooms in Italy do not meet optimal listening conditions. Furthermore, the reasons for students’ poor listening quality appear to not be fully understood, neither by students nor by teachers. In addition to the need for greater attention to physical learning spaces (advocating the universal design principles), effective change will also need to involve a greater awareness of what the barriers to listening are and how much they influence both teaching and learning quality and effectiveness.

## 1. Introduction

Education in schools predominantly relies on two sensory modalities, vision and auditory perception, with the latter being pivotal in contemporary pedagogical approaches. Multiple research projects have consistently shown that uncorrected vision adversely affects education, as shown by Basch [1], indicating that the ideal remedy is to rectify the visual impairment to enhance academic achievement. Most also noted that hearing loss adversely affects educational outcomes [2].

Three primary factors affect the quality of listening in educational settings: classroom acoustics, individual hearing abilities, and the teachers’ vocal delivery.

First of all, we emphasize the significance of the importance of an appropriate acoustic environment in an educational context. Crandell and Smaldino [3] demonstrated that the learning process, reliant on effective communication and the comprehension of verbal messages, can be hindered by the physical/environmental conditions of classrooms, typically marked by elevated background noise and excessive reverberation. A review conducted by Mealings indicated that suboptimal classroom acoustic conditions, including diminished signal-to-noise ratios, elevated noise levels, and prolonged reverberation times significantly adversely affect university students’ listening, learning, and well-being [4]. A study by Astolfi et al. demonstrates that prolonged reverberation periods, linked to inadequate classroom acoustics that result in elevated noise levels and diminished speech intelligibility, lead to a decreased perception of enjoyment and self-happiness among students [5]. Conversely, in a separate review, Mealings also examined the role of teachers, aiming to comprehend the impact of classroom acoustic conditions on their health and well-being. The findings indicated that elevated noise levels or an increased number of students adversely affect instructors’ health and well-being [6]. The issue of inadequate classroom acoustics is exacerbated in students with hearing impairments (a partial or total inability to hear sounds in one or both ears); for this category of students, the listening conditions in the classroom must be particularly favorable. Indeed, the World Health Organization, regarding auditory capability, indicated in its 2021 report that hearing loss can significantly affect an individual’s educational achievements. Without prompt assistance, individuals with hearing loss experience poorer academic performance, an increased potential of school dropout, and a reduced probability of pursuing higher education in comparison to their hearing counterparts [7]. Also, Khalsa et al. in their cross-sectional study noticed that hearing impairment status may be the key factor directly associated with differences in school involvement [8]. As previously mentioned, the voice of teachers is essential in the learning process. A speaker’s compromised voice quality can hinder speech intelligibility, particularly in noisy settings [9].

As suggested by the literature, it is widely recognized that educators’ vocalizations experience disorders; a recent meta-analysis estimated the prevalence of voice disorders among university teachers at 41% [10]. Schiller et al. revealed that exposure to hoarseness was associated with increased perceived listening effort, impaired cognitive performance, and increased displeasure [11]. This factor, along with previously examined issues such as inadequate acoustics in classrooms and impaired hearing, contributes to subpar academic achievement.

Overall, these aspects have been extensively examined in primary schools but somewhat less so in secondary schools and colleges, where listening likely assumes an even more critical part in the learning process. Pupils in higher education must comprehend and analyze progressively complex concepts, making the clarity of these essential [11].

The newly implemented UNI 11532-2 [12] in 2020 is a technical standard that delineates the acoustic criteria for educational settings, in contrast to UNI 11532-1 [13] in 2018, which addressed constrained spaces more broadly. This technical standard delineates the threshold values for characteristics like reverberation time (T), speech transmission index (STI), and clarity index (C50), tailored to the requirements of schools. Moreover, UNI 11532-2 offers comprehensive guidelines for the acoustic design and assessment of educational environments, taking into account measurement uncertainty and the use of sound-absorbing materials. It is also mentioned in the Minimum Environmental Criteria, rendering its compliance obligatory in public procurement concerning school buildings [14].

This article presents the findings of a survey conducted as part of the A.Ba.Co. project (Overcoming Communication Barriers), promoted by the Friuli-Venezia Giulia region, Italy, and funded by the Office for Disabilities of the Presidency of the Italian Council of Ministers.

The ABaCo project addresses the issues encountered by individuals with hearing impairments. These conditions impose both physical and sensory limits on individuals and may also result in socio-cultural barriers over time. In the absence of adequate support, these obstacles can negatively impact individuals’ quality of life and restrict social inclusion [15]. A.Ba.Co. strives to create inclusive educational environments that provide communication, participation, and accessibility. The initiative acknowledges and emphasizes the distinct requirements and characteristics of students or individuals with hearing impairments. The project aimed to ensure comprehensive access to communication and understanding in schools for all types of hearing and communication disabilities. It attempted to combine technological innovations with a flexible approach to address the diverse needs of students with varying degrees of deafness or hearing impairment. The project’s initial milestone planned an online questionnaire aimed at describing students’ and teachers’ perceptions and definitions of their hearing and auditory experiences in the classroom.

## 2. Materials and Methods

We conducted a qualitative study by administering questionnaires. Two surveys were created as part of the A.Ba.Co. (Overcoming Communication Barriers) initiative, initiated by the Friuli-Venezia Giulia Region and financed by the Office for Disabilities of the Presidency of the Council of Ministers. A questionnaire was directed to secondary school students in Friuli-Venezia Giulia and at students of the University of Perugia, as well as another questionnaire aimed at secondary school teachers in Friuli-Venezia Giulia and at teachers of the University of Perugia (Table 1). In the Friuli-Venezia Giulia region, the following schools were involved: the Liceo Classico F. Petrarca of Trieste, the Istituto Superiore—G. Leopardi-E. Majorana of Pordenone, the Istituto Superiore—Magrini Marchetti of Udine, and the Istituto Superiore—Michelangelo Buonarroti of Monfalcone. For the University of Perugia, all faculties were involved.

Closed or open-ended questions were used to examine the replies of students and teachers concerning diagnosed or perceived hearing impairments in daily life, the quality of auditory perception in classroom settings, and the potential causes for diminished hearing clarity. This study examined teachers’ perspectives regarding students’ listening experiences and their own vocal effort during instruction. Responses were analyzed between those with hearing impairments and those without, as well as between students and teachers throughout the two higher education levels. The surveys were disseminated via email to university students and faculty, while for secondary schools, they were transmitted by email to teachers and through an electronic register to students and their parents. In most participating schools, teachers administered the questionnaire in class to actively engage the students during school activities.

The survey instrument aimed to investigate two primary points: 1. to assess the prevalence of clinically certified and perceived hearing difficulties among students and faculty and 2. to determine the quantity and placement of students with hearing disabilities across university departments.

The survey was indeed designed to explore three additional areas in greater depth: 1. an examination of the university experience for students with hearing sensory disabilities, incorporating perspectives from both students and faculty; 2. the perception and impact of acoustic aspects on students and faculty in the learning process; and 3. strategies employed by lecturers regarding their teaching practices.

The questions through which the items were articulated were identified based on both national and international studies reviewed in the context of the A.Ba.Co. project, as well as the strategic analysis conducted by the A.Ba.Co. team. Furthermore, given the absence of targeted studies on auditory sensory disability within the university demographic in Italy, it was deemed necessary to conduct an in-depth investigation of the subject through focused interviews with the FIADDA (Associated Italian Families for the Defense of the Rights of Hearing-Impaired People) Association. A specific analysis of the regulations and organizational management of pathways for students with disabilities was conducted at the University of Perugia.

All questions analyzed in this study were categorical variables, so data are reported as number and percentage, and between-group comparisons were made using Chi-square test or Fisher’s exact test where appropriate. The significance threshold was set at a *p*-value of 0.05. Analyses were performed using Stata17 [16].

## 3. Results

A total of 2228 responses were collected from students and 378 from teachers across four high schools in the Friuli-Venezia Giulia region and the University of Perugia, Italy.

Four educational institutions of Friuli-Venezia Giulia collaborated, comprising a total of 3314 students and 428 teachers. A total of 1141 students participated in the questionnaire, resulting in a participation rate of 34.43%. Regarding the teachers involved in the project, 154 out of 428 teachers responded, resulting in a participation rate of 35.98%.

A total of 27,382 questionnaires were distributed via email to the personal addresses of students enrolled in first- and second-level courses during the university academic years 2021 and 2022. A total of 1087 students responded to the questionnaire, resulting in a response rate of 3.97%.

In relation to the teaching staff, 1939 newsletters were distributed, resulting in 224 responses to the questionnaire, which corresponds to a response rate of 11.55%.

### 3.1. Students

Among university students, the most represented departments were Philosophy (6.2%), Medicine and Surgery (3.49%), Chemistry (1.93%), Literature, and Political Sciences (1.28%). Hearing difficulties, either clinically diagnosed or only perceived, were reported by 8–9% of students. Concerning auditory impairments, it was revealed that 2.11% (23/1087) university students possessed a documented hearing difficulty, whereas 6.43% (70/1080) experienced hearing difficulties without a formal diagnosis (Table 2).

A total of 0.84% (9/1064) secondary school pupils reported a confirmed hearing loss, while 7.33% (78/1064) suggested hearing impairments without certification (Table 3).

A hearing impairment correlates with increased challenges in classroom comprehension for students [7]. In response to the question, “During the lesson, do you sometimes miss the content entirely or partially?”, 16% indicated that they frequently or always encounter difficulties in understanding the teachings. Nonetheless, when students were queried regarding the audibility of the teacher’s lecture, no statistically significant disparities were observed between students with normal hearing and those with stated hearing impairments. In reply to the inquiry, “Do you feel, or have you felt the necessity for the teacher to present material at a slower pace?” 25% stated that this need occurred frequently or consistently. Of these, just 15 possessed an actual hearing impairment. The occurrence of hearing impairments seems to exhibit a modest correlation with the necessity for the educator to decelerate their presentation (*p* < 0.001). There is a significant difference between individuals without hearing difficulties and those with hearing difficulties without a clinical diagnosis (t (−3.929), *p* < 0.001).

Among the students who answered the question “When in class, can you hear well the lecturer’s explanations?”, the predominant reported reason for inadequate listening difficulties in class varied across the two student groups; 45.8% (488/1065) of high school students identified noise in the room as the cause, whereas 18.2% (86/472) of university students did so (*p* < 0.001). Conversely, 40.9% (193/472) of university students attributed their listening difficulties to their position in class, compared to 9.5% (101/1065) of high school students (*p* < 0.001) (Table 4).

### 3.2. Teachers

An analysis of the hearing profiles revealed that between teachers, the reported hearing difficulties were 27.1% (39/144) in high school and 12% (27/224) at university. A statistically significant difference was seen between secondary school teachers and university teachers with hearing difficulties, favoring the former group (*p* < 0.001) (Table 5).

The majority of the teachers in both school grades, when asked about their own perceived tone of voice during teaching, deemed the students’ listening experience (Table 6) to be good or better despite claiming to use a high tone of voice and perceiving vocal fatigue (Table 7).

When asked “How often do you need to raise your voice to be heard clearly?”, a statistically significant difference was found between secondary school teachers and university teachers. The former showed a greater need to raise their voice than the latter (*p* < 0.001) (Table 8).

The inquiries concerning vocal effort indicated a statistically significant disparity between secondary school and university teachers regarding the frequency of reported voice strain. The data reveal that a higher proportion of secondary school instructors experience vocal strain compared to university educators (*p* < 0.0001). The necessity for teachers to raise their vocal volume seems to be correlated with the perceived quality of the auditory environment (*p* < 0.001).

## 4. Discussion

A.Ba.Co. seeks to create inclusive educational environments that ensure communication, participation, and accessibility. The project identifies and emphasizes the distinct requirements and characteristics of students or individuals with and without hearing disabilities. The questionnaire was developed to describe the perceptions and definitions of hearing and auditory experiences among students and their teachers. It investigates the listening challenges encountered by both groups at the University of Perugia and in four secondary schools in Friuli-Venezia Giulia, Italy.

An interesting initial finding from these questionnaires was the extensive prevalence of hearing difficulties among the student population, exceeding those reported to the Disability Office of the University of Perugia (seven students from the initial analysis). In our sample, 23 students had a certified hearing disability, while an additional 70 reported undiagnosed hearing difficulties. The data concerning certified hearing disabilities for the four secondary schools is unavailable to us. However, in this group of students, 9 were clinically diagnosed with hearing difficulty, while 78 were found to have no hearing impairment despite reporting hearing difficulties in class. The overall presence of difficulties, whether diagnosed or perceived, was comparable between the two groups of students, suggesting a potentially similar epidemiology. Recent Istat data on disability indicate that in the 2020/2021 high school classes, 3.02% of the student population was affected by some form of disability, with 2.3% specifically representing hearing disabilities [17]. At the university level, recent data indicate that among the 1.82% of students with disabilities, 6% had a hearing disability [18]. In addition, among university educators, there were 5 with clinically certified hearing disabilities compared to 22 out of 224 who had non-clinically certified hearing difficulties. Secondary school teachers report a higher prevalence of hearing disabilities, with 7 teachers having certified hearing impairments and an additional 32 out of 144 experiencing non-certified hearing difficulties. We found a statistically significant difference between secondary school teachers and university teachers with hearing difficulties, favoring the former group (*p* < 0.001). The age of the teachers was assessed to rule out the possibility that one of the two groups had older instructors; however, the data did not yield significant results. It is interesting to note that clinically unidentified hearing difficulties occur frequently among teachers, as they do for students. Numerous individuals report difficulties with hearing; however, there is inadequate reporting of these issues to school authorities. Richardson et al. reported that only 8% of postsecondary students in the United States with hearing loss disclosed this condition to their institutions [19]. A recent study by Tajima, Ishikawa, Fujioka, and Murata (2024) indicates that school-age students increasingly experience listening difficulties as they age, particularly concerning auditory attention and memory, highlighting the importance of incorporating self-reports for the early identification of concealed listening problems [20]. As a supplementary observation (not included in the results but posed as a question in the questionnaire), the survey indicated that, in total, only 20 out of 32 students with documented hearing loss had utilized hearing aid accommodation (either a hearing aid or cochlear implant), whereas merely one out of seven teachers acknowledged employing any form of technology to address their hearing impairment, despite a number of teachers with hearing loss indicating they had received a formal diagnosis of the condition. These results are associated with the characterization of hearing loss as a “invisible disability.” This designation arises not only from the absence of visible symptoms but also from the longstanding stigma within communities and neglect by policymakers [7].

Hearing loss in children, adolescents, and adults is often associated with feelings of inadequacy and diminished self-esteem. Individuals with hearing loss, even when managed, often exhibit the stigma linked to hearing impairment and the utilization of hearing devices [21]. Conversely, not all individuals who report hearing issues actually have a hearing impairment. A comprehensive analysis of the data suggests that “perceived” hearing issues may not solely constitute an individual medical concern but are also influenced by environmental barriers to effective listening, such as noise levels in high schools and seating arrangements in university classrooms. Consequently, it may indeed be a “functional” issue, which would partially elucidate why most difficulties are merely seen. Our study revealed that among students responding to the question “When in class, can you hear the lecturer’s explanations clearly?”, the primary cited cause of hearing difficulties differed across the two student groups; 45.8% (488/1065) of high school students cited ambient noise as the cause, while 18.2% (86/472) of university students did the same (*p* < 0.001). Conversely, 40.9% (193/472) of university students ascribed their listening challenges to their seating arrangement, in contrast to 9.5% (101/1065) of high school students (*p* < 0.001). To support this theory, we present the noteworthy testimony of a university student who utilizes a wheelchair due to his disability. The student indicated no hearing issues; however, they struggle to hear effectively in the classroom due to the absence of a ramp that would allow them to approach the teacher and thereby improve their auditory experience. This illustrates a case of perceived hearing difficulty resulting from an architectural barrier. This testimony illustrates that contemporary solutions cannot solely rely on the concept of “barrier-free environments” which traditionally involves constructing or modifying buildings, structures, and transportation to accommodate individuals with physical disabilities, such as the installation of wheelchair ramps.

It is essential to move beyond the notion of “without barriers” to adopt a contemporary understanding of universal design. This international term encompasses a modern and comprehensive design methodology aimed at creating buildings and environments that are inherently accessible to all individuals, regardless of the presence of a disability. Today, we should prioritize designing for ease of access and usability from the outset. These concepts have been shown to be highly beneficial and, in many instances, crucial for usability among non-disabled individuals experiencing temporary disabilities, as well as for various situations and needs, including specific conditions of fatigue, stress, and adverse environmental circumstances [22].

In this context, the teachers’ replies are noteworthy, as they not only seem to validate the environmental “obstacle” to hearing but also underscore a deficiency in knowledge and, subsequently, a lack of understanding regarding the need of effective listening among students in class. The same educators indicated that the listening experience in class for pupils was rated as good or very good (Table 6); nevertheless, they subsequently noted a necessity to elevate their vocal tone, which was significantly higher among high school teachers (*p* < 0.001) (Table 7). This data is associated with that concerning the auditory challenges faced by students, revealing that high school students regarded classroom noise as the primary cause of these difficulties. It is reasonable to assume that the noise levels in high school classrooms are considerable, resulting in increased auditory challenges for students and necessitating louder speech from teachers, in contrast to the university setting.

### Limits

The response rate from university students was quite low (3.97%); nevertheless, it is important to note that the emails were dispatched to all specified addresses, disregarding the existence of registered but inactive students. Moreover, it should be considered that individuals with clinically certified hearing impairments might have responded more, as they relate closely to the subject matter. The small sample size may diminish the statistical power of this analysis and restrict the applicability of these findings to the wider university student population. Furthermore, it is important to note that the data concerning patients with clinically diagnosed hearing loss is based exclusively on the survey, as no audiological tests were performed.

## 5. Conclusions

The findings from the questionnaire directed at students and teachers, concentrating on higher education and the Italian educational context, reveal a notable incidence of listening difficulties among both high school and university students and educators, with a considerable proportion indicating perceived issues that did not attain a clinical diagnosis.

Students with hearing impairments report a higher incidence of acoustic disturbances compared to their normally hearing counterparts and face greater challenges; however, our data reveal that a significant number of students experience diminished listening clarity linked to environmental factors, including classroom noise and seating arrangements. The elements contributing to these issues vary between high school and university, indicating the necessity for customized strategies to meet the distinct needs of students at different educational levels.

Teachers’ feedback on their assessment of students’ auditory perception and the volume of their typical classroom voice indicates a likely deficiency in knowledge and awareness of the aspects that significantly contribute to inadequate listening in the classroom.

These observations underscore the finding that the potential solutions align with the principles of universal design, which prioritizes the creation of accessible and inclusive environments for all individuals, particularly those with varied learning needs and abilities, including teachers, as they aid in alleviating strain and vocal load. Universal design can be practically applied in classroom settings through the implementation of noise-reducing materials to enhance acoustic quality, adjustable layouts to facilitate mobility and visibility, multi-format instructional materials (such as text, audio, and video), and flexible assessment methods that cater to diverse learning needs while maintaining educational objectives.

## Figures and Tables

**Table 1:** Attributes of the questionnaires designed for teachers and students.

Teachers	Students
52 closed or open-ended questions	50 closed or open-ended questions
10–15 min are required for completion	10–15 min are required for completion
Analyzed areas: Listening experience (both in classroom settings and distance learning) Vocal effort, importance of the lesson in the learning process Experience with a student with hearing difficulties Distribution and characteristics of auditory impairments	Analyzed areas: Listening experience (both in classroom settings and distance learning) Distribution and characteristics of auditory impairments

**Table 2:** Results (number of respondents, percentage) of the following question asked to university students: “What kind of difficulties do you encounter?” NH = normal hearing; HI = hearing impairment.

University	HI	NH
	**N = 23**	**N = 70**
**What kind of difficulties do you encounter?**	*n* (%)	*n* (%)
Speech discrimination	5 (21.7)	16 (22.8)
Unilateral hearing loss	10 (43.5)	17 (24.3)
Bilateral hearing loss	8 (34.8)	5 (7.1)
Tinnitus	4 (17.4)	10 (14.3)
Sound distortions	2 (8.7)	14 (20.0)
Excessive annoyance from loud noises	2 (8.7)	26 (37.1)
Others	1 (4.3)	8 (11.4)

**Table 3:** Results (number of respondents, percentage) of the following question asked to high school students: “What kind of difficulties do you encounter?” NH = normal hearing; HI = hearing impairment.

High School	HI	NH
	**N = 9**	**N = 78**
**What kind of difficulties do you encounter?**	*n* (%)	*n* (%)
Speech discrimination	2 (22.2)	14 (17.9)
Unilateral hearing loss	4 (44.4)	18 (23.1)
Bilateral hearing loss	2 (22.2)	24 (30.8)
Tinnitus	2 (22.2)	11 (14.1)
Sound distortions	1 (11.1)	7 (9.0)
Excessive annoyance from loud noises	2 (22.2)	40 (51.3)
Others	0	6 (7.7)

**Table 4:** Complete results (number of respondents, percentage) for the question “When in class, can you hear well the lecturer’s explanations?”. Among the university students, this inquiry pertained solely to students who participated in person at a university. NH = normal hearing; HD = hearing difficulties.

“When in Class, Can You Hear Well the Lecturer’s Explanation?”						
	High School Students			University Students		
	Total 1065	NH	HD	Total 472	NH	HD
Yes, always	472 (44.3%)			183 (38.8%)		
No, never	4 (0.4%)	4 (0.8%)	0	8 (1.7%)	5 (2.0%)	3 (6.7%)
It depends on wehere i’m sitting	101 (9.5%)	83 (15.9%)	18 (25.7%)	193 (40.9%)	169 (67.0%)	24 (53.3%)
It depends on the noise in the classroom	488 (45.8%)	436 (83.3%)	53 (74.3%)	86 (18.2%)	76 (30.2%)	10 (22.2%)
Oter	0	0	0	2 (0.4%)	2 (0.8%)	0

**Table 5:** Results (number of respondents, percentage) of the following question asked to high school and university teachers: “What kind of difficulties do you encounter?”.

Teachers	High School	University
	**N = 39**	**N = 27**
**What kind of difficulties do you encounter?**	*n* (%)	*n* (%)
Speech discrimination	10 (25.6)	7 (25.9)
Unilateral hearing loss	19 (48.7)	7 (25.9)
Bilateral hearing loss	8 (20.5)	9 (33.3)
Tinnitus	7 (17.9)	8 (29.6)
Sound distortions	3 (7.7)	2 (7.4)
Excessive annoyance from loud noises	10 (25.6)	6 (22.2)
Others	1 (2.6)	3 (11.1)

**Table 6:** Complete results (number of respondents, percentage) of the following question asked to teachers: “From your point of view, the students’ listening experience is”.

Teachers	High School	University
	**N = 144**	**N = 224**
	*n* (%)	*n* (%)
Insufficient	6 (4.2%)	5 (2.2%)
Sufficient	29 (20.1%)	20 (8.9%)
Good	60 (41.7%)	62 (27.7%)
Very good	40 (27.8%)	106 (47.3%)
Great	9 (6.2%)	31 (13.9%)

**Table 7:** Complete results (number of respondents, percentage) of the following question asked to teachers: “When teaching, you think you use”.

Teachers	High School	University
	**N = 144**	**N = 224**
	*n* (%)	*n* (%)
A low tone of voice	6 (4.2%)	4 (1.8%)
A normal tone of voice	67 (46.5%)	132 (58.9%)
A loud tone of voice	71 (49.3%)	88 (39.3%)

**Table 8:** Complete results (number of respondents, percentage) for the following question asked to university and high school teachers: “How often do you need to raise your voice to be heard clearly?”.

Teachers	High School	University
	**N = 144**	**N = 224**
**How often do you need to raise your voice to be heard clearly?**	*n* (%)	*n* (%)
Never	10 (6.9)	54 (24.1)
Rarely	33 (22.9)	68 (30.4)
Some time	57 (39.6)	62 (27.7)
Almost always	35 (24.3)	35 (15.6)
Always	9 (6.3)	5 (2.2)

## Data Availability

Data is available upon request.
